# Is the Level of Motor Development at School Entry Related to the Use of Municipal Exercise Programs? A Social-Differential Analysis

**DOI:** 10.3390/ijerph19053047

**Published:** 2022-03-05

**Authors:** Daniel M. Faßbender, Katharina Kreffter, Simon Götz, Maurus Hagemeister, Stefanie Lisak-Wahl, Thuy Ha Nguyen, Theodor Stemper, Simone Weyers

**Affiliations:** 1 Faculty of Medicine, University Hospital Duesseldorf, Centre for Health and Society, Institute of Medical Sociology, Moorenstrasse 5, 40225 Duesseldorf, Germany; simon.goetz@uni-duesseldorf.de (S.G.); maurus.hagemeister@uni-duesseldorf.de (M.H.); simone.weyers@uni-duesseldorf.de (S.W.); 2 Hamm-Lippstadt, University of Applied Sciences, Marker Allee 76-78, 59063 Hamm, Germany; katharina.kreffter@hshl.de; 3 Academy of Public Health Services, Kanzlerstrasse 4, 40472 Duesseldorf, Germany; lisak-wahl@akademie-oegw.de; 4 IGES Institute, Friedrichstrasse 180, 10117 Berlin, Germany; thuy-ha.nguyen@iges.com; 5 School of Human and Social Sciences, University of Wuppertal, Sport Science, 42119 Wuppertal, Germany; stemper@uni-wuppertal.de

**Keywords:** promoting physical activity, motor skills, physical benefits, prevention, municipal exercise programs, social inequality, school entry health examination, preschool children

## Abstract

Children’s motor development is socially unevenly distributed despite many municipal exercise programs (EXP). It has not been sufficiently investigated whether and how they appeal to children from different social backgrounds. This study investigates the use of municipal EXP in preschool age and the association between participation and motor development considering social circumstances. In school entry health examinations, parents were asked about participating in various EXP (response = 65.5%; *n* = 6480). Motor development, i.e., body coordination and visual-motor coordination, were assessed by a social pediatric development screening, and social circumstances by migration background (MB) and parental education (PE). Poisson regression estimated adjusted Incidence Rate Ratios (IRR; 95% confidence interval, 95%—CI) for relationships between social circumstances and participation in programs and participation and body coordination/visual-motor coordination. Children with MB (IRR 0.73; 95%—CI 0.71–0.75) and low PE (IRR 0.45; 95%—CI 0.40–0.50) used EXP less often. Children participating less often have a finding in body- (IRR 0.76; 95%-CI 0.63–0.90) and visual-motor coordination (IRR 0.47; 95%—CI 0.35–0.62). Significant effects were found for children with and without MB and higher PE. Municipalities should make EXP more attractive for families with MB and low PE.

## 1. Introduction

Physical activities and the reduction of sedentary behavior are essential for successful development in children. Especially in early childhood, physical activity (PA) promotes not only the physical, but also the mental, emotional, and psychosocial development with corresponding consequences for their health [1,2,3]. Children with increased PA have better motor development [1,2,4], which can promote the formation of a positive self-concept and also better acceptance in social structures [3,5]. Poorly developed motor performance because of little PA causes deficient development in the areas of motor skills (especially body coordination), posture, and obesity and a lower acceptance in the social environment [6].

Motor skills are defined as the entirety of all control and functional processes on which posture and movement are based [7]. Posture and movement are therefore considered as visible phenomena and motor skills, also referring to psychomotor and neuromotor skills, as their cause [7]. If a skills-oriented approach to motor research is followed, motor skills can be differentiated to describe and explain individual motor performance [5]. According to Bös et al. [5], a distinction is first made between conditional and coordinative skills. On level two, a distinction is made between the basic characteristics of endurance, strength, speed, and coordination. On level three, 10 motor sub-skills are located, such as aerobic and anaerobic endurance, high-speed, and maximum strength or the speed of action and reaction, based on training parameters such as duration, scope, and intensity. The development of motor skills understood as theoretical constructs can be assessed with the help of motor tests with defined movement tasks [5,8]. In school entry health examinations, motor performance is often assessed based on coordinative skills, differentiated into coordination under time pressure (e.g., jumping back and forth for 15 s) and coordination in precision tasks (e.g., balancing).

The prevalence of a developmental coordination disorder in children of preschool age (3–7 years) amounts between 5–10% in studies in European countries [9,10,11]. Worldwide, even higher prevalences have been observed, which, however, could be associated to the use of different methods [12]. Nevertheless, a lack of body coordination skills is one of the most common developmental delays among school newcomers [13]. Coordinative performance development shows clear social inequalities. Socioeconomically disadvantaged children, regardless of their age, do worse in tests of standing long jump, swimming, and cycling [13], and show poorer results in hand-eye coordination, stance balance, trunk mobility, and endurance [14]. Furthermore, they are worse positioned in terms of their overall body composition and endurance performance than their classmates with a higher socioeconomic status [8,15]. In addition, children with migration backgrounds (MB) show worse results in terms of motor performance than their peers without MB [5,16].

There are many municipal exercise programs (EXP) to promote the motor development of children. Whether they succeed or not has not yet been sufficiently investigated. A review by Venetsanou et al. [17] shows that the participation of 2–6-year-old children in physical education programs in daycare centers or schools is associated with better motor development. Röhr-Sendlmeier et al. [18] and Röhr-Sendlmeier [1] report on comparable results. Children from a psychomotor-oriented kindergarten, who, however, also had a higher level of recreational sports activity, demonstrated significantly better motor performance than children from a conventional kindergarten. A limitation their studies have in common is that the participation in EXP is mostly linked to a membership in facilities such as daycare centers and schools and is not open to everyone. Municipalities are, furthermore, central settings because they create opportunities and spaces that encourage people to be physically active. These include residential environments, parks, leisure and sports facilities. In addition, the framework for structured PA, i.e., interventions that are guided by adults and are intended to promote motor development and the enjoyment of movement, is created in the municipality [19]. 

Overall, there is little evidence on the promotion of PA for children in the leisure setting [3]. Rethorst [20] observed slight differences in motor performance after membership in a sports club in 3–7-year-old children. Wirszing [21] examined the effects of sports leisure activities in primary school children and found that activities directly related to sport and movement (e.g., in sports clubs) promote motor development. To the best of our knowledge, a differentiated study of the benefits of various EXP in the leisure area has not yet been carried out. In specialist publications, there is only consensus that children should move in as many and diverse ways as possible and reduce sitting times as much as possible [3,22].

In addition, there is the problem that socioeconomically disadvantaged children and children with MB, and therefore especially those with higher educational needs, take advantage of prevention and health promotion programs less often [23,24]. This also applies to the area of PA promotion, which could be demonstrated especially for older children [21,25] but also occasionally for children who are just starting school [26,27]. 

Finally, there is a lack of evidence regarding the effects of social differences in interventions to promote PA. It is still unclear which groups will benefit from the measures [28]. A more differentiated analysis according to the types of offer and social circumstances for the age group of preschool children would be expedient in order to identify supply gaps in municipal prevention for this important developmental period in children’s motor coordination. 

In view of the research gaps, the aim of this study is to examine whether the use of various municipal EXP in preschool age is related to the level of motor development when entering school. Special attention is paid to the social circumstances of the children, and it is examined whether EXP reach children from educationally disadvantaged families and those with MB and whether they are associated with better body- and visual-motor coordination. Representative survey data in the context of the school entry health examination allow the analysis of various EXP taking social differences into account. By that we would like to answer the following questions:

How often are various municipal EXP used by children in preschool age considering their different social circumstances?

Is there a connection between the use of municipal EXP during preschool age and motor development at the time of the school entry health examination for children in different social circumstances?

## 2. Materials and Methods

### 2.1. Study Design

The data were collected as part of the retrospective cohort study, “Healthy on school entrance” [29]. Between October 2016 and August 2018, parents of school beginners in the municipality of Duesseldorf were surveyed in the school entry examination with a standardized written questionnaire assessing the use of municipal prevention offers from birth to school entrance. The survey data were linked to the medical examination results. With a response rate of 65.5%, 6480 children were included in the study, of whom both the parental questionnaires and the medical examination results were available. To answer the study question, all participants were included for whom the necessary variables were complete. This resulted in a number of *n* = 5461 subjects with whom the analyses were continued.

The variables underlying the analysis were measured as follows: the use of EXP was surveyed for the categories parent-baby course up to 1 year, parent-child course between 1 and 6 years, exercise and play group without parents, and daycare center with a focus on exercise and swimming course (yes/no in each case). 

A finding in the area of motor development was obtained using the two areas of body coordination and visual-motor coordination of a social pediatric development screening (SOPESS) [30]. SOPESS is a validated screening instrument developed for school beginners and used comprehensively in several German states. Body coordination was assessed by jumping back and forth in order to measure, e.g., balance skills, endurance, and dosage of strength. Visual-motor coordination was assessed by two drawing tasks (geometric shapes and recreating two motifs) in order to measure, e.g., hand-eye-coordination, fine motor skills, and recognizing forms and figures [31]. According to a point score given for each task, the doctor classifies the developmental status in the following six categories. The categories A (clarification of the findings by a registered doctor necessary) and X (findings that do not require treatment) were compared with category K (no findings). Children who were already in therapy (B), children with disabilities or permanent impairments (D), and children for whom the examination could not be carried out (O) were excluded from the analyses. 

Measuring the social situation: a migration background (MB) was assumed if at least one parent was born abroad [32]. Parental education (PE) was recorded on the basis of school and vocational education and based on the CASMIN (Comparative Analysis of Social Mobility in Industrial Nations) classification differentiated between parents with low vs. parents with medium/higher education [33]. 

### 2.2. Statistical Analysis

The use of the various types of offers was initially determined for the total sample and then stratified by MB and PE (absolute and relative frequencies). The statistical significance of the group differences was calculated using the chi-square test. The association of MB and low PE with the use of the various offers was analyzed in multivariate models using Incidence Rate Ratios (IRR) with 95%—confidence intervals (CI) by means of Poisson regression, with the age and gender of the child and the respective other indicator of social circumstances being adjusted. The association of the use of EXP with motor development (i.e., body coordination and visual motor coordination) was also determined by a multivariate basis, initially for the total sample (adjusted for the age, gender, MB, and education level of the parents), then stratified by MB and by PE (adjusted for age, gender, and the respective other indicators of social circumstances). 

The analyses were carried out with the statistics program STATA 14.2 (StataCorp LLC, College Station, TX, USA). 

## 3. Results

In the sample (Table 1), 2769 children are male (50.7%). The mean age is 5.46 years. Of the children, 11.1% have a finding (categories A or X) in body coordination, 4.8% in visual-motor coordination, 52.3% have an MB, and 10.7% have parents with a low level of education. 

Overall, 72.8% of the parents surveyed reported that their children had used an EXP. The differentiated analysis of the individual EXP shows that 52.2% of the children had taken part in swimming programs (such as baby swimming or swimming class), 45.7% in exercise and play groups without parents, and 44.2% in parent-baby courses. Of the children, 29.8% had taken part in a parent-child course. Of the parents surveyed, 6.6% stated that their children had been to a daycare center with a focus on PA (Table 2).

Social situation and use of municipal EXP: analyzing the social differences, it shows that both children from a family with a migrant background and children from a family with a low PE had used almost all offers significantly less than their reference group. Of the children with MB, 59.9% had used one of the offers, as compared to 86.9% of the children without it. Regarding the PE, the difference is 32% for children with low PE as compared to 77.6% for children with medium/high PE. 

In the multivariate model, too, the Incidence Rate Ratios (IRR) show that children with MB are less likely (IRR 0.73; 95%—CI 0.71–0.75) to have participated in an EXP than children without MB. The same applies to children from families with lower PE compared to children with higher PE (IRR 0.45; 95%—CI 0.40–0.50). The largest differences according to MB can be found in the parent-baby course (IRR 0.42; 95%—CI 0.40–0.46) and in the parent-child course (IRR 0.49; 95%—CI 0.45–0.53). The greatest differences according to the PE can be found in parent-child courses (IRR 0.12; 95%—CI 0.08–0.20) and swimming programs (IRR 0.19; 95%—CI 0.15–0.25). There are no significant correlations in daycare centers with a focus on PA.

Municipal EXP and motor development: for the overall sample, it was shown that children who took part in EXP have a 0.76-fold (95%—CI 0.63–0.90) probability of a finding in body coordination compared to the children who did not participate (Table 3). In the consideration of the individual programs, the strongest correlation was shown for participation in swimming programs (IRR 0.69; 95%—CI 0.58–0.82). No significant correlation can be observed in the parent-child course and in the daycare center with a focus on PA.

Taking social differences in consideration of the connection between use of a program and the level of motor development, it shows both for children with (IRR 0.75; 95%—CI 0.62–0.91) and without MB (IRR 0.46; 95%—CI 0.35–0.60) a lower probability of a finding in body coordination in connection with the general use of an EXP. This also applies to the individual parent-baby courses, exercise, and play groups without parents and swimming programs. In the parent-child course, an IRR 0.68 (95%—CI 0.53–0.88) only shows a significant correlation for children without MB. Daycare centers with a focus on PA are still not associated with a lower probability of a finding in either group. 

The stratification for the education level of parents shows that both children from families with low PE (IRR 0.69; 95%—CI 0.48–0.99) and children from families with medium/higher PE (IRR 0.74; 95%—CI 0.61–0.89) have a lower probability of a finding in body coordination in connection with the use of an EXP. This also applies to parent-baby courses (IRR 0.57; 95%—CI 0.28–0.93 or IRR 0.70; 95%—CI 0.59–0.83). Daycare centers with a focus on PA are still not significantly associated with a lower probability of a finding in either group. In addition, exercise and play groups without parents (IRR 0.71; 95%—CI 0.60–0.85) and swimming programs (IRR 0.67; 95%—CI 0.57–0.80) are only associated with a lower likelihood of a finding in body coordination in children with medium/high PE.

Table 4 shows the results of the analyses regarding the relationship between the use of the programs and findings in visual-motor coordination, both for the total sample and then stratified according to MB and PE. In the overall sample, the general use of an EXP is also associated with a lower probability of a finding in visual motor coordination (IRR 0.47; 95%—CI 0.35–0.62). This also applies to the individual types of programs, again with the exception for the daycare center with a focus on PA.

This is also evident when the analyses are carried out and stratified according to MB, both for general use (IRR 0.38; 95%—CI 0.28–0.51 for children with and IRR 0.26; 95%—CI 0.17–0.40 for children without MB) and for the individual types of EXP. However, if the PE is considered separately the result is different: in children from families with medium/high PE, the general use and the use of the individual types of programs were significantly associated with a lower probability of a finding. Even with children from families with low PE, the probability of a finding in visual-motor coordination is reduced with the general and individual use of programs, but the correlation is not statistically significant. 

## 4. Discussion

The aim of this study was to investigate how often different municipal EXP are used in preschool age by children in different social circumstances. It was also examined whether there is an association between the use of municipal EXP in preschool age and motor development at the time of the school entry health examination in children considering different social circumstances. 

In summary, it was found that around three-quarters of the children took part in an EXP, most often in swimming programs, exercise and play groups without parents and parent-baby courses. Daycare centers with a focus on PA were rarely used. A differentiated analysis showed that children with MB participated less often and children from families with lower PE participated far less often than their respective reference group. When considering the MB, the greatest differences were found in the use of parent-baby courses and parent-child courses. The largest differences in PE were found in parent-child courses and swimming programs. 

The social inequalities found here correspond to the few studies on the use of PA in the leisure sector among younger children. Finger et al. [34] show that children with a low socioeconomic status meet the WHO recommendations [22] regarding PA to a lesser extent. This is supported by Poulain et al. [35] and Cook et al. [36] reporting on less PA and lower levels of motor performance skills in children with low socioeconomic status. A study by Brophy et al. [26] also shows that children from socioeconomically better-off families participate more often in organized sports activities and children of Asian or African descent do so less often. Additionally, Ahmed et al. [37] underlined in their recent systematic review that children with MB show less PA than their reference group. However, no distinction was made between the types of programs being used. Wirszing [27] examined indicators of sports activity in a more differentiated way. It was also shown that children from families with a higher social status participated more frequently in offers of the club sports sector. 

While most studies focus on the general use of EXP, we examined the use of various municipal programs in the leisure sector in preschool age because the municipality contributes to child development and health with several offers and facilities [38]. Two results stand out particularly, helping to answer questions about the causes of social inequalities in the use of EXP. The biggest problems in reaching target groups are (a) parent-baby courses for children with a migrant background and (b) swimming programs for children from families with low PE.

Therefore, social barriers could be assumed in the first place. In parent-baby courses, for example, social interactions involve more intimacy than other EXP in different age groups. In addition to the physical proximity of parents and babies, it is also explicitly about parents making new social contacts. The different everyday practices, preferences, and attitudes of the sociocultural groups that are discussed in connection with the dilemma in prevention could possibly have an impact here. Franzkowiak [39] assumes that by means of the neglect of the everyday practices of disadvantaged groups, their distance to educational and preventive care offers is reinforced. Second, there are also economic barriers to participating. Swimming programs are often more cost intensive than EXP offered by the local gymnastics and sports clubs. Based on parent surveys, costs have already been identified as a common barrier to sport for children [40]. By a cost analysis, it was now possible to calculate that EXP for children from socioeconomically disadvantaged families are not always affordable. Although non-profit organizations already grant a number of discounts for families in difficult circumstances, swimming programs in particular take up a large part of the freely disposable income of a family on social welfare benefits [41]. 

The present study also shows that general participation in PA is associated with a lower likelihood of a finding in body coordination and visual-motor coordination. However, there are differences according to the type of program, social circumstances, and area of development. In the area of body coordination, the association applies especially to parent-baby courses, exercise and play groups without parents and swimming programs, mostly regardless of the children’s social circumstances. In the area of visual-motor skills, the association applies to almost all EXP (especially in swimming programs), independent of the MB. Regarding the PE, the results are only significant for children from families with medium/higher PE. In contrast, there is no statistically significant association for children from families with low PE. This could be caused by the small sample size of children from families with low PE. However, it could also be the case that EXP are less effective in this group.

The connection between the use of a program and development could be causally justified by the fact that extra movement in the first years of life is an essential component in the motor development of children because children learn “comprehensively” at the beginning and therefore open their world in a variety of ways [42]. Various studies have already shown that participating in EXP has a positive effect on the motor development of children. For the leisure sector, Rethorst [20] and Wirszing [27] demonstrated that young children who take part in club sports performed better in motor tests than their peers who did not. Wrotniak et al. [43] found a positive correlation between PA and visual-motor development. However, the latter assumed that good motor development favors enjoyment and therefore participation in sport, which constitutes a reverse causal relationship. However, due to the cross-sectional study design, the authors could not answer this question conclusively. 

### Limitations

As a limitation of our study, it must be said that the retrospective study design, in which parents had to provide information on the past 6 years, involves methodological problems. Parents must remember which programs they have made use of with their children in the last 6 years. When interpreting the results, it must also be considered that no reliable statement can be made about causality. In addition to the problem of cause and effect described above, it was not possible to measure other factors in (early) childhood that could affect both participation in PA and motor development. Finally, measuring motor development with a focus on coordination, as is common in school entry health examinations, is limited. As mentioned in the introduction, motor development encompasses other dimensions that are difficult to describe fully in a routine examination. On the other hand, a survey within the framework of the school entry health examination has great potential: it is compulsory for all school newcomers [44], whereby a high recruitment rate can be achieved and children from families that are difficult to reach can also be included [29]. In this way, the question of the benefit of various EXP can be viewed in a large sample taking different social circumstances into account. Linking the parent’s survey results with the data of the medical examination also makes it possible to determine the endpoints of motor development based on validated [30] examinations.

Taking these limitations into account, it can initially be concluded that the access routes to EXP for families with MB and those with low PE should be expanded. This includes spatial and economic access: setting approaches aim to reduce such spatial barriers to health promotion. Children and young people seldom leave their district, so sports and EXP must be implemented in their living area in order to enable low-threshold access, especially for socially disadvantaged children. It is essential to consider the interests and wishes of the people living in the respective district and to include local structures and networks such as daycare centers, schools, and sports clubs. In intervention studies, “health scouts” have proven to be extremely helpful. They assess the needs of the district’s residents and make it easier for them to access municipal EXP [45,46]. Also, economic barriers should be reduced. The municipal parties involved should improve the financial access to offers that encourage PA by further optimizing prices for socially disadvantaged families. EXP are not easily affordable for families with financial difficulties [41]. There are good indications that financial incentives have a positive effect on participation in prevention or “lifestyle programs” [47,48], even with low-income parents [49]. 

Furthermore, the results suggest that targeted interventions may be necessary for children with low PE. Presumably, these children have many risk factors regarding motor development, so that individual single programs are not sufficient to ensure good motor development. These risk factors depend on the individual lifestyle, such as high media usage [6,50], up to environmental factors such as poor mobility in the district [6,51]. In a low-stimulus environment, various structural interventions are necessary, such as measures to improve traffic safety, playgrounds and parks, and leisure and sports facilities [3]. This is important because children of preschool age have a pronounced need for movement and every suggestion for their independent activity is meaningful. At the behavioral level, parent-baby courses could play an important role, as they can teach young parents important attitudes and competencies in this context. Parent surveys show that they value the time in parent-baby courses as a meaningful experience for themselves and their babies, as much as the information and suggestions [52]. In the present study, children of various educational and ethnic backgrounds benefited from parent-baby courses. Nevertheless, it is assumed that prevention programs often take into account the everyday practices of socio-cultural groups insufficiently [39]. Parent-baby courses should therefore be further developed and evaluated regarding the specifics of different groups.

## 5. Conclusions

In view of the frequency of motor development delays among young children, the promotion of PA in the municipality should have a high priority. In view of the social inequalities both in motor development and in the use of EXP, new ways should be developed in which children from families with low PE and children with MB can be made aware of offers, motivated to participate, and supported by target-group-specific offers. Early childhood EXP could play an important role here.

## Figures and Tables

**Table 1:** Sample Description: Observations (*n*) and Percentages (%) or Mean and Standard Deviation (SD).

	Categories	*n* or (Mean)	% or (SD)
Age		(5.46)	(0.26)
Gender	Male	2769	50.7
	Female	2692	49.3
Body coordination finding	Yes ^a^	607	11.1
	No ^b^	4854	88.9
Visual-motor coordination finding	Yes ^a^	261	4.8
	No ^b^	5200	95.2
Migration background	Yes	2854	52.3
	No	2607	47.7
Parental education	Low	584	10.7
	Medium/high	4877	89.3
Total		5461	100.0

^a^ Children with a finding and need of clarification by a registered doctor (category A) or no need of clarification (category X); ^b^ Children with no finding (category K).

**Table 2:** Use of Exercise Programs for the total Sample and according to Social Situation.

Offer Type	Use of the Entire Sample	Use according to Migration Background			Use according to Parental Education		
		No		Yes	Medium/High		Low
	*n* (%)	*n* (%)		IRR (95%—CI) ^a^	*n* (%)		IRR (95%—CI) ^b^
Exercise programs in general	3973 (72.75)	2265 (86.88)	1708 (59.85)	0.73 (0.71–0.75)	3786 (77.63)	187 (32.02)	0.45 (0.40–0.50)
Parent-baby course up to 1 year	2415 (44.22)	1691 (64.86)	724 (25.37)	0.42 (0.40–0.46)	2362 (48.43)	53 (9.08)	0.24 (0.17–0.31)
Parent-child course 1 to 6 years	1625 (29.76)	1097 (42.08)	528 (18.50)	0.49 (0.45–0.53)	1605 (32.91)	20 (3.42)	0.12 (0.08–0.20)
Exercise and play group without parents	2493 (45.65)	1432 (54.93)	1061 (37.18)	0.73 (0.69–0.77)	2406 (49.33)	87 (14.90)	0.33 (0.27–0.40)
Day care centre with a focus on exercise	359 (6.57)	180 (6.90)	179 (6.27)	0.92 (0.75–1.12)	325 (6.66)	34 (5.82)	0.90 (0.64–1.27)
Swimming programs	2852 (52.22)	1771 (67.93)	1081 (37.88)	0.61 (0.58–0.65)	2796 (57.33)	56 (9.59)	0.19 (0.15–0.25)

Incidence Rate Ratios with 95% confidence interval for participation in the offer; ^a^ Reference category no migration background, adjusted for low parental education, age and gender; ^b^ Reference category medium/high parental education, adjusted for migration background, age and gender.

**Table 3:** Incidence Rate Ratios for a Finding in Body Coordination.

Participation in:	Total Sample ^a^	According to Migration Background		According to Parental Education	
		Yes ^b^	No ^b^	Low ^c^	Medium/High ^c^
	IRR (95%—CI)				
Exercise programs in general	0.76 (0.63–0.90)	0.75 (0.62–0.91)	0.46 (0.35–0.60)	0.69 (0.48–0.99)	0.74 (0.61–0.89)
Parent-baby course up to 1 year	0.71 (0.60–0.86)	0.73 (0.57–0.93)	0.56 (0.44–0.71)	0.57 (0.28–0.93)	0.70 (0.59–0.83)
Parent-child course 1 to 6 years	0.90 (0.75–1.09)	0.96 (0.75–1.24)	0.68 (0.53–0.88)	0.63 (0.22–1.80)	0.87 (0.73–1.05)
Exercise and play group without parents	0.74 (0.62–0.87)	0.72 (0.58–0.88)	0.60 (0.47–0.77)	0.78 (0.49–1.25)	0.71 (0.60–0.85)
Day care centre with a focus on exercise	0.95 (0.69–1.30)	0.95 (0.63–1.44)	0.94 (0.57–1.54)	0.80 (0.38–1.71)	1.00 (0.70–1.41)
Swimming programs	0.69 (0.58–0.82)	0.65 (0.52–0.80)	0.56 (0.44–0.71)	0.60 (0.31–1.67)	0.67 (0.57–0.80)

Incidence Rate Ratio (IRR) with 95% confidence interval for findings in body coordination; ^a^ Reference category no participation; adjusted for age, gender, migration background, parental education; ^b^ adjusted for age, gender, parental education; ^c^ adjusted for age, gender, migration background.

**Table 4:** Incidence Rate Ratios for a Finding in the Visual-Motor Coordination.

Participation in:	Total Sample ^a^	According to Migration Background		According to Parental Education	
		Yes ^b^	No ^b^	Low ^c^	Medium/High ^c^
	IRR (95%—CI)				
Exercise programs in general	0.47 (0.35–0.62)	0.38 (0.28–0.51)	0.26 (0.17–0.40)	0.74 (0.48–1.13)	0.35 (0.26–0.47)
Parent-baby course up to 1 year	0.53 (0.38–0.75)	0.52 (0.35–0.77)	0.31 (0.20–0.48)	0.85 (0.42–1.72)	0.44 (0.32–0.61)
Parent-child course 1 to 6 years	0.54 (0.37–0.78)	0.55 (0.35–0.86)	0.29 (0.17–0.52)	0.56 (0.15–2.09)	0.48 (0.33–0.71)
Exercise and play group without parents	0.53 (0.39–0.71)	0.38 (0.26–0.55)	0.47 (0.30–0.73)	0.61 (0.32–1.15)	0.48 (0.38–0.66)
Daycare center with a focus on exercise	0.55 (0.29–1.04)	0.64 (0.30–1.34)	0.38 (0.09–1.51)	1.12 (0.52–2.42)	0.28 (0.09–0.87)
Swimming programs	0.30 (0.21–0.42)	0.27 (0.18–0.41)	0.20 (0.12–0.31)	0.52 (0.22–1.22)	0.27 (0.19–0.37)

Incidence Rate Ratio (IRR) with 95% confidence interval for findings in the visual-motor coordination; ^a^ Reference category no participation; adjusted for age, gender, migration background, parental education; ^b^ adjusted for age, gender, parental education; ^c^ adjusted for age, gender, migration background.

## Data Availability

The data analyzed during the current study are not publicly available because the data owner is the City of Duesseldorf.
